# Cocooning Nurse Autonomy in Türkiye: Navigating a Path to Professionalism That Does Not Challenge Medical Dominance

**DOI:** 10.1111/1467-9566.70196

**Published:** 2026-05-06

**Authors:** Zuleyha Inceoz, David Hughes

**Affiliations:** ^1^ Department of Nursing Carroll College Helena Montana USA; ^2^ Faculty of Medicine, Health & Life Science Swansea University Swansea UK

## Abstract

Nursing work in several Western countries has been affected by evolving discourses of managerialism and professionalism. Interdisciplinary working has given nurses more prominence in high‐level teams and created hybrid management roles that have affected understandings of professionalism. Such changes generally followed broader new public management (NPM) reforms that shifted power from senior doctors to executive managers. Yet, although there is an extensive literature on the global spread of NPM reforms, less is known about the influence of associated discourses concerning nurse management and professionalism. This paper addresses that gap by presenting qualitative data on the evolving situation of hospital nursing in Türkiye, a country that implemented NPM‐type reforms in the early 2000s. Based on 40 in‐depth interviews completed in 2021/22, it describes the uneven impact of these reforms on medicine and nursing, the continuing reality of medical dominance and the development of a professionalising project among Turkish hospital nurses that avoids directly challenging medical power. This emphasises continuing professional education, practice guideline development and a curtailed form of teamwork away from doctors. Nurses exercised greatest autonomy in specialised wards, intensive care units and emergency departments, where a stable staff group could operate at a distance from oversight by senior doctors.

## 1 Introduction

This paper considers how far discourses of managerialism and professionalism that have emerged largely in Anglophone and Scandinavian countries have influenced the development of hospital nursing and nursing management in Türkiye. Recent social science literature from Western countries describes a constellation of changes centring on interdisciplinary teamwork, distributed leadership, hybridity and shifting professional identities that is changing the nature of interprofessional relations and nursing's position vis‐à‐vis other disciplines (Nancarrow and Borthwick 2005; Traynor 2009; O'Reilly and Reed 2011; Fitzgerald et al. 2013; Waring et al. 2020). Nursing in many countries is being reshaped through interprofessional working and the recruitment of nurses into a wider range of management roles. Decisions on matters such as case management and care plans are increasingly being made in multiprofessional teams rather than by medical professionals acting alone. Moreover, the emergence of hybrid management roles occupied by nurses brings them into closer interaction with general managers (Croft et al. 2015a). Yet, although these developments affecting nursing's professional identity receive considerable attention from researchers in Western countries, we need to ask whether such changes amount to a global trend. The question arises as to whether changes like those reported in the UK literature are starting to emerge in healthcare systems outside the West, where control by medical elites is largely unchallenged.

Our research examined the situation of nurses and nurse managers in Türkiye, an upper‐middle‐income country that straddles Europe and the Middle East, which has nevertheless been influenced by Western healthcare reform models. The wider study investigated nurses' perceptions of the changing nature of nurse management, nursing work and interprofessional relations and the opportunities for and barriers to professional development. Overall, the study found that opportunities for Turkish nurses to move into hybrid management roles of the kind reported in the United Kingdom were virtually nonexistent and that the exercise of greater nursing autonomy via increased influence in interdisciplinary teams or work away from medical control was very limited. This paper focuses on the narrower issue of how nurse managers and staff nurses develop their practice in the shadow of continuing medical dominance. It provides a useful point of contrast with recent literature on Western countries by describing how Turkish nurses have embarked on a distinctive professionalisation path, adapted to a context in which traditional management structures and medical power are still strong.

### 1.1 Managerialism, Professionalisation and the Reordering of Interprofessional Relationships

It can be argued that the two key movements affecting the future position of the nursing profession are managerialism and professionalism. Although the two movements often stand in tension, there are also times when they can support each other. Each offers a route to improve nursing's position vis‐à‐vis other disciplines. Managerialism creates a path for professional advancement by allowing senior nurses to join high‐level management teams and break free of traditional hierarchies and because it may weaken the position of medical professionals who have traditionally controlled the healthcare division of labour (Numerato et al. 2012). At the same time, nursing continues to strive to improve its professional standing by building a distinctive knowledge base and marking out spaces for autonomous practice (Walby and Greenwell 1994; Traynor 2009). In several Anglophone and Scandinavian countries, the development of standardised care pathways and managed clinical networks has resulted in wider recognition of a separate nursing input. Nancarrow and Borthwick's (2005) review charts the rise of interdisciplinary teamwork and its impact on the healthcare division of labour and occupational boundaries (see also Liberati 2017). Nurses are represented in greater numbers in high‐level teams, with managers from health disciplines other than medicine playing a part in decision‐making (Fitzgerald et al. 2013; Waring et al. 2020). Several commentators point to a corresponding shift from the traditional ‘concentrated’ leadership associated with formal line‐management position to distributed leadership involving persons with appropriate expertise from multiple disciplines and across different institutional levels (McKee et al. 2013; Fitzgerald et al. 2013).

The increasing importance given to interdisciplinary teamwork needs to be set in the context of longer‐term changes in management structures. In many healthcare systems, administration overseen by doctors has been transformed, first by the rise of general managers and then by notions of executive leadership (O’Reilly and Reed 2011; Martin and Learmonth 2012; Erskine et al. 2013; Smith et al. 2018). These changes were usually associated with wider new public management (NPM) reforms (Dunleavy and Hood 1994) intended to improve health system efficiency. The discourses surrounding management and leadership continue to evolve, shaped in part by the increasing involvement of nurses in management roles (Learmonth 2019).

A key development that has stirred interest in the social science literature is the emergence of hybrid managers who combine managerial and clinical roles, initially mainly medical professionals but increasingly also nurses and allied health professionals (Burgess and Currie 2013; Waring et al. 2020). This brings nurse managers into closer contact with core management activities such as resource allocation and performance management and may raise difficult questions concerning occupational identity (Bresnen et al. 2019).

There are tensions between the hard‐edged concerns of resource management and the idea of nursing as a holistic caring discipline (Newman and Lawler 2009). Moreover, hybrid nurse managers must adjust to changed relations with doctors and the historically gendered nature of senior management (Croft et al. 2015a). Croft et al. (2015b) argue that the type of transformational leadership that might appeal to nurses is less common than a more transactional form of managerialist leadership concerned with day‐to‐day performance management. These authors argue that nurse leaders try to manage this tension through ‘identity work’, at times distancing themselves from a managerial leader identity.

Changes that result in hybrid management also result in forms of hybrid professionalism (McGivern et al. 2015; Noordegraaf 2015). Greater involvement in management may support, rather than impede, the development of a nursing knowledge base and nursing's jurisdiction over certain areas of practice. Part of the work of hybrid nurse managers in the United Kingdom and Scandinavian countries like Denmark is oversight of standards and guidelines linked to quality improvement, thereby mediating between managerial and professional hierarchies (Spyridonidis and Currie 2016; Ernst 2019). Traynor (2009) argues that nursing has drawn on evidence‐based medicine to aid guideline development and expand its knowledge base, but at the cost of reducing the indeterminacy that is a hallmark of autonomous professional practice. This aligns with Noordegraaf's (2015) argument that hybridisation results in a reorganisation of professional work to combine contradictory professional and managerial principles, such as autonomy and control or quality and efficiency. Professionals in management roles often ‘balance or blend’ the two frameworks (McGivern et al. 2015, 412), bringing softer elements of managerial discourses into their understanding of professional work.

Although these trends are visible in the United Kingdom and certain other Western healthcare systems, we should note that debate continues about whether, despite official policy and surface changes, medical elites find ways to retain power. UK research on managerial oversight of general practice doctors suggests a reordering, rather than disappearance, of medical power, with senior professional peers often playing a mediating role (Grant et al. 2015). Sheaff et al. (2003, 625) described how general managers passed responsibility for enforcing rules to local professional leaders who used a form of ‘soft governance’ to achieve ‘a gradual introduction of managerial techniques’ in primary care. Regarding the wider healthcare system, Waring et al. (2020) suggest that English NHS healthcare networks develop novel forms of hierarchy in which ‘network elites’—mainly senior doctors in hybrid professional/management positions—retain substantial power to shape network policy and service configurations. In a similar vein, Jones et al. (2016) found that directors of nursing on Welsh health boards faced continuing problems in overcoming the power of established medical and managerial hierarchies.

Overall, the spread of interdisciplinary teamwork, hybrid management roles and distributed leadership in these Western countries has changed the surface nature of interprofessional relations, raised the professional standing of nursing and provided a path that might be attractive to nurses in other countries, even if nursing gains coexist with a subtle reordering of doctor power. To date, few similar developments in middle‐ and lower‐income countries have been reported, but the question arises as to how far these may gain traction, especially where other NPM‐type reforms have been introduced. As we will outline shortly, nurse managers in Türkiye are eager to develop both nurse professionalism and nurse management, but, although cognisant of Western reform discourses, are navigating a novel path tailored to their own situation.

### 1.2 Nursing and the Health Transformation Programme

Türkiye is one of several middle‐income countries that emulated the NPM reforms that reshaped the public healthcare systems of many Western countries (Agartan and Kuhlmann 2019). The Health Transformation Programme (HTP), introduced in phases from 2003, created a purchaser/provider split with a single central purchasing agency and (over time) merged the existing health insurance schemes into a single state general health insurance scheme that brought free universal healthcare coverage (Polin et al. 2022). The changes included a major restructuring of public hospitals, the creation of over 20 large public/private partnership healthcare complexes known as city hospitals, the introduction of various efficiency incentives such as short‐term employment contracts and performance‐related supplementary pay and a brief experiment with nonmedical executive hospital managers.

The reforms had an uneven impact on the different health professions, especially regarding the contrasting fortunes of medical practitioners and nurses. The original HTP reform design was opposed by many doctors because it appeared set to weaken the position of medical elites by introducing general managers and putting hospital directors on short‐term contracts. But within a few years, these reform elements were watered down to return to the situation where all hospital directors were medically qualified, effectively leaving traditional medically dominated management structures in place.

Nursing did much less well. There was a move to all graduate entry to the profession, but this did not significantly disturb an existing order in which many nurse managers trained in old‐style nursing colleges had risen to senior positions through seniority, trade union links or patronage. A more significant change involved the replacement of nursing directorates with health service directorates in MoH and city hospitals. This generally left senior nurses in a new healthcare service director (or ‘health service manager’) role but made them responsible for managing a broader group of nonmedical hospital professionals and services. Critics argued that this effectively removed the nursing line‐management structure and reduced the attention that health service directors could give to core nursing work. There was also dissatisfaction with the limited rewards going to nurses under the performance‐based supplementary payment system (PBSPS) that replaced pay based solely on standard salary scales. Although doctors received a salary supplement directly related to the units of clinical activity completed in a month, the more modest payments going to nurses were based on an ‘incentive coefficient’ calculated for their hospital work unit and largely unrelated to the content or volume of work undertaken by individual nurses.

This left a feeling in Turkish nursing circles that the reforms had diminished the influence of nursing as a separate professional discipline and blurred the boundaries between the work of nursing and other nonmedical healthcare occupations. A recent report from Türkiye's main nursing representative body demands changes to correct current problems: a ‘predictable single‐item salary’ rather than a split between basic salary and PRSPS payments; a review of the health service directorate structure and return to the ‘matron model’; ending the expectation that nurses will undertake tasks outside their job description; ending the delegation of nursing duties to other professions; ensuring that management appointments are based on merit; and creating ‘an independent structure’ to represent nursing within the Ministry of Health (MoH) under the name ‘Department of Nursing’ (Turkish Nursing Association (Türk Hemşireler Derneği) 2023).

The aim of the wider study on which this paper is based was to assess how far Türkiye has moved along a pathway towards interprofessional team working in hospital care that gives a significant role to nurses. The key research questions addressed included the following: What is the current situation of nurse management in Turkish hospitals, including the perceived problems and the facilitators of and barriers to change? How do nurses perceive the meaning of leadership and the role of nurse managers in hospitals? How far has nurse management emulated certain developments, such as interdisciplinary working, hybrid management and team decision‐making, observed in certain Western countries? This paper touches on the above questions and moves on to consider how far the changes that have occurred in nurse management and understandings of professionalism have affected the exercise of nurse autonomy.

## 2 Method

These questions were investigated through a qualitative study that utilised individual in‐depth interviews with 40 hospital nurses. Participants were 20 staff nurses and 20 nurse managers, including 14 in senior management positions. The respondents were spread across two MoH public hospitals, two university hospitals and a public–private partnership ‘city’ hospital, reflecting the main public‐sector hospital types in Türkiye. All the hospitals were in the Istanbul area. The qualitative interview method was chosen to allow respondents to describe the situation and ongoing changes in Turkish nursing management in their own words, explaining the context of hospital nursing work as they saw it and so permitting the researcher to explore aspects of nurses' accounts that might have been missed by closed‐end survey questions. Ethical approval for the study was gained from research ethics committees at Swansea University, UK, and Marmara University, Istanbul. The Executive Nursing Association (*Yönetici Hemşireler Derneği*), a national professional body, was approached to seek support for the study and help with contacting nurses for interviews. The association responded positively and provided a list of potential respondents in the Istanbul area, which had been selected as the research site to make the study manageable with the limited resources available.

The 40 participants were essentially a convenience sample with purposive elements. Initial interviews were completed with a small number of nurses suggested by the Turkish Executive Nursing Association, and these respondents then provided names of other potential recruits (snowball sampling). These were contacted to assess their willingness to participate, and selections were made to achieve a spread of grades, participant characteristics and hospitals. The aim was to achieve an even split of managers and frontline nurses, including a spread of ages and some males (a small minority in the study hospitals). Recruits needed to meet the inclusion criteria of possessing at least a bachelor's degree and minimum 5 years of hospital work experience (experience that would enable them to reflect on ongoing changes in nursing work). The possibility of extending the sample was left open should 40 participants prove to be insufficient to achieve data saturation, but in the event, a judgement was made that there were enough overlapping interview accounts to confirm the picture emerging, and fieldwork ended when 40 interviews were concluded. Subsequently, when knowledge gaps emerged regarding a few matters of detail, a small number of informants were recontacted by telephone or email to gather missing information.

Semi‐structured interviews were conducted between September 2021 and February 2022. The interviews took place using the Zoom application because of travel restrictions during the COVID‐19 pandemic. The interactions typically lasted between 60 and 90 min and were recorded with the subject's permission using the facility within the Zoom application. The primary questions centred on the changing state of nursing management in Türkiye, the extent to which this was influenced by ideas from the literature about nurse leadership, distributed leadership, interdisciplinary teamwork and hybrid roles, barriers and facilitators of change and ideas about how nurse management could be improved. This paper focuses mainly on what nurses said about professional development, nurse leadership, interprofessional relations and the constraints affecting nurse autonomy.

The recorded interviews were fully transcribed and subjected to thematic analysis. Although the study was influenced by Glaser and Strauss's (1967) method of constant comparative analysis, the analysis focused more on the identification and examination of key concepts and themes than on theory building (as in conventional grounded theory). The approach is not dissimilar to the generic form of thematic analysis recommended by Braun and Clarke (2006), but with more emphasis on a continuing process of comparing, coding and recoding fragments of data.

As a first step, the transcribed passages were analysed and coded question by question, acknowledging that broad themes implied by the interview questions might yield answers addressing other major themes and subthemes. Each question file, comprising responses from all participants to a specific question, was read and reread to identify patterns and subthemes. Generally speaking, a cluster of themes and subthemes emerged in answers to a particular area of questioning, so that comparison of responses in a given area was helpful in determining how subthemes fitted together. Passages identified for potential use were translated by the authors. These were given codes and a number to indicate how many instances of passages illustrating that subtheme had been found. This first‐order coding allowed the generation of an initial list of subthemes within the data, and we were then able to compare these with other responses across the whole dataset. In many cases, a theme resurfaced under later questions so that theme categories and codes were refined further throughout the exercise. For more detail of the study methodology, see Inceoz (2024).

### 2.1 Medical Dominance and Interprofessional Relationships

It quickly became apparent from the interviews that Turkish nursing is traversing a very different path from that portrayed in the Anglophone literature mentioned earlier. Doctors still play a dominant role in the management of hospitals, opportunities for interdisciplinary teamwork are limited, and hybrid clinical/professional roles in which nurses have real power are virtually nonexistent, including vis‐à‐vis the new health service director role. As one nurse health service manager explained, the medical profession dominates every level of the Turkish healthcare system.

Türkiye has a physician‐dominated healthcare management system. Everything is physician‐oriented in our system. For instance, the Minister of Health is a doctor, the deputy ministers are doctors, all the top health system managers are doctors. Only the financial, administrative or similar departments are not managed by doctors. We are the backbone of the health service: nurses, and nurse managers. But we do not have any place in the top health service management structure. For instance, there are committee meetings regarding the revolving *funds* for nurses [the PRSPS *funding* source]. Everybody is there but no nurse manager. Nobody to represent nurses.

(M5‐F‐MoH Hospital)^1^

MoH rules require that all hospital directors (also known as head physicians) be medically qualified. A new head physician typically brings in their own senior management team, and this creates a patronage network that shapes relations between team members.

The head physician has very real power over the executive nurses. Every time the head physician changes, when the director of the hospital changes, unfortunately, he comes with his own team. He or she brings in nursing staff of their own. This nursing manager may not have any management training. There are even those who have graduated from vocational school and studied at the Open University; then they became managers such as in our hospital. There is no selection based on valuing people's experience or education, the one closest to management is chosen.

(N5‐F‐City Hospital)

This was perceived to undermine the separation of nursing and medical management hierarchies. Rather than having a manager from the appropriate discipline overseeing a given hospital division, as in the previous system, where there were designated medical and nursing service directors, the current management structure allows doctors to exercise control across the whole organisation. Although organisational plans and management nomenclatures vary somewhat between hospital types, a typical arrangement might involve a chief physician operating as deputy to the head physician, at the same level as a finance director, a support and quality director and a healthcare service director, and then, at the next level, a number of senior doctors heading speciality‐based subdirectorates. Apart from finance and healthcare services, all senior positions may be held by medically qualified professionals, all having more power than the healthcare service director.

Of course, there will be a head physician, but the fact is that other hospital directorates are also led by physicians and we are always obliged to give them an explanation. In other words, physicians can play a very active role in planning, training, employing or maintaining order in the clinic here. For example, a nurse who disagrees with the clinic chief physician can be replaced. But a physician is never replaced, the nurse is replaced. This is usually a situation that arises due to the fact that the autonomy lies with the physician.

(DM2‐F‐University Hospital)

Although healthcare service managers in the study were all nurses, the role has been widened to include oversight of professions allied to medicine, with the consequence that line‐of‐command control over frontline nursing became more complicated.

I see it this way, there was an expansion of responsibilities. The healthcare services manager (…) is now the manager of health professionals. The scope of their duties has expanded. But it seems to me that her time and authority in dealing only with nurses has decreased. How can I describe this? Her time and duties are divided into a thousand pieces. There are a lot of responsibilities. She just cannot do her main job.

(DM4‐F‐City Hospital)

A key tenet of Freidson's (1970) notion of professional dominance is that the medical profession has the ability not only to define the content of its own work but also to influence and control the division of labour with other health professions. Although nursing exerts increasing control over the content of nursing work in Western countries, a recurrent complaint of nurses in this Turkish study was that doctors were able to go too far in allocating tasks to nurses that did not fall within their formal job description. Several nurses expressed frustration because, notwithstanding the duties and responsibilities set out in hospital regulations, nurses found themselves ordered to pick up mundane tasks that were the doctor's responsibility.

The doctors do not even write their drug orders; they have the nurse to write them instead. Nurses do a lot of work that doctors should be doing. In fact, the law determines our duties and responsibilities, but it is not implemented.

(N16‐F‐University Hospital)

There was a problem regarding discharge in our department. The junior doctors order all the necessary materials that will be used in a surgical operation from the hospital pharmacy, they use it, and the operation is finished. Then they ask us to enter details of the medical consumables and equipment used in the operation in the system. If we don’t enter the information, the patient can’t be discharged

(N18‐F‐University Hospital)

The redrawing of professional boundaries in Western countries, as, for example, when UK nurses took on expanded roles in the early 1990s, generally involved the delegation of technical tasks or new areas of discretion that promised to raise nurses' professional standing (Allen and Hughes 1993). However, even when new roles were on offer, many nurses complained that this could also involve the transfer of undesirable tasks from a higher to a lower occupational group (Allen 2000), a process described in the classic sociology of professions literature as passing down ‘dirty work’ (Hughes 1951). The majority of Turkish study participants saw the work pushed their way very much in these terms, complaining that doctors were handing them tedious, time‐consuming chores demeaning to nurses.

For several participants, medical ‘*hegemonya*’ (hegemony) was the underlying issue that lay behind current problems. Some saw this as an intractable problem rooted in a wider society, which continues to be organised around patriarchal values that shape gender roles in the home and workplace and are ingrained in medical and nursing cultures.

There is physician hegemony. The physician is always dominant. I find this very wrong. The physician decides everything. Why? Why does the physician decide? Why is that? (…) I think this is due to the structure of society. (…) First it is culture and then there is the structure of Turkish society.

(DM4‐F‐City Hospital)

Medical dominance reduces the space in which senior nurse managers can exercise power and the degree of autonomy in nursing practice. Many participants bemoaned the inability of senior nurses to attain senior executive positions and the limited influence they had as nursing professionals. The absence of a separate, autonomous chain of command within nursing, resulting from the HTP reforms, was a source of resentment. This was a product both of medical power and the phasing out of head nurses in favour of healthcare service managers.

Although nursing seems to be a separate discipline, a separate management, and a separate department, unfortunately on paper in many places, the authority of physicians is still above nursing. This inevitably narrows down your management area.

(M3‐F‐University Hospital)

If we don't interfere with the medical administration, they shouldn't interfere with us. In fact, at this level of the organisation the physician should be completely separate.

(DM1‐F‐University Hospital)

For these nurses, it seemed unfair that the dominant medical hierarchy remained largely untouched by recent health reforms, whereas nursing had lost the separate line‐management structure that they believed necessary for the efficient oversight of nursing work.

The negative consequences of medical dominance have been well described in the Anglo‐American literature, but an additional factor in Turkish hospitals is that doctor power is enacted in a system where doctors, nurses and other health professionals are civil servants. This means that care must be delivered within a framework of bureaucratic rules that impede autonomous nursing practice and change and within hierarchies that are shaped by political allegiances and connections as much as professional merit. Not only are certain aspects of human resource management and career progression affected by civil service regulations but also this has a deep effect on the culture of the service, so that, for example, the possibilities for instigating nursing practice innovations through bottom‐up initiatives are very limited.

We manage here according to the law and regulations, that’s it. (…) We have a civil servant mentality; people do not make any effort and get paid. So, nobody cares about innovation and change. It is difficult to convince people, I mean top managers. We cannot even convince nurses to join some seminars, they directly ask whether they’ll get paid, why would they come otherwise … waste of time according to them.

(M5‐F‐MoH Hospital)

Of course, we have the civil service system. This is a big obstacle to being a leader. Every employee is an officer, and nobody can make anyone do anything because there are not very strict rules to discipline a civil service officer. This is a very bureaucratic environment.

(DM6‐F‐University Hospital)

Historically, doctors have held the senior positions in civil service hierarchies in the health sector so that the regulations and chain of command do not limit their professional practice to the same extent as for nurses. The constraints that limit the ability of nurses to develop autonomous practice therefore tend to support the existing structures underpinning medical dominance.

Even for those managers who work to the best of their ability, it is hard to ignore the civil service culture that affects others around them. Among other things, informants alleged that poorly performing civil servants have little possibility of dismissal and therefore little incentive to do more than the minimum at work.

There are also nurses who are not competent in this way. For example, we can't get these nurses out of the job. Unfortunately, we can't get them out of the job because they are civil servants. (…) So okay, there's discipline and scrutiny, but it doesn't extend to sacking. It is a big problem that incapable and poorly educated nurses are working in hospitals.

(M2‐F‐University Hospital)

This widespread criticism of the drawbacks of civil service employment is consistent with the findings of research from political science and health policy on Turkish public administration more generally. Research from the 2000s paints a picture of public services characterised by patronage, political partisanship, corruption and excessive bureaucracy (Sözen and Shaw 2002; Acar and Emek 2008). More recent work suggests that limited improvements in governance have been offset by a crisis arising from ‘over‐politicisation’ (Akkoyunlu 2018; Üstüner and Yavuz 2018). Between 2016 and 2021, over 130,000 civil servants, including doctors, linked to the Gülenist movement and other groups opposed to the Erdoğan government, were removed from their posts (HM Government 2025).

### 2.2 The Segmentation of Nursing and Struggle to Advance Professionalism

The claims by some nurses that colleagues are ‘incapable and poorly educated’, that ‘nobody cares about innovation and change’ and that nursing is not an ‘organised’ professional group point to divisions in the nursing workforce. Although outside the scope of this paper, one finding of the study was that four professional segments were visible among hospital nurses (Inceoz 2024). Participants who favoured further nursing reform divided into two groups. A handful were attracted to mechanisms such as audit and performance management that had been central to the wider HTP reforms and might be termed the *bureaucratic modernisers*. Others who supported reform pinned their hopes on developing a nurse leadership role that stressed improved patient care, evidence‐based practice and continuing professional education. This group can be labelled the *professional modernisers*. Both moderniser groups believed that progress was held back by a minority of colleagues who did not share their vision. These colleagues were nurses wedded to an existing system that had brought them advancement or job security (the *civil service traditionalists*) and those who adopted an instrumental orientation to nursing work, regarding it as a steady job rather than a vocation (the *pragmatists*).

This paper focuses on the largest group in the study, the *professional modernisers*, their vision of nursing leadership and their strategy for advancing their agenda. Their aim was to transform nursing from within rather than to gain greater decision‐making authority at the higher levels of management controlled by doctors. When participants talked about these hopes in the interviews, they tended to focus upon a few key areas—team building, professional education, evidence‐based practice and nursing standards development—which were largely under their control and less dependent on the support of medical patrons. The words they used included ‘quality patient care’, ‘innovative’ practice, ‘team’ work, ‘solidarity’, ‘evidence‐based’ practice and a move towards ‘multidisciplinary’ working.

We have a corporate culture that is open to innovative education, good patient care, quality patient care. We also give importance to quality. A ‘let me insert the serum, let me sit down to work’ style does not exist in our hospital. What is important for us is to give good care to our patients, to give quality care. There is also solidarity among the staff, although sometimes there are small problems. We work as a team along nursing lines. We are moving towards multidisciplinary working.

(S2‐F‐University Hospital)

Some modernisers define their position by describing the old‐style practices they want to avoid.

There are a lot of factors that influence you to be a nurse leader ‐ family, educational factors, cultural structures, intellectual structures. (…) All of this makes nurses less interested in science; the nurse does not read, does not research, does not develop herself. Therefore, when she becomes a manager, she becomes a manager with the same structures, with little existing knowledge. A docile group become rulers who take their power from the regulations, you know, from the legislation. The number of nurses who do not have a vision, a purpose, but just want to have a job, is very high. In order to be a leader, it is necessary to read, to be curious, to have different characteristics.

(DM1‐F‐University Hospital)

Others stressed the importance of experience and showing willingness to get involved in frontline nursing work, whether to set an example or to demonstrate that they were not just office‐bound managers who gave orders from afar.

A nurse who has not held a patient's hand, cannot be an educator, I have great doubts about how a nurse who has not managed a clinic or who has not done patient management can be a good manager. This means that without clinical experience, you cannot take an active role in nursing education or management.

(S3‐F‐University Hospital)

This discourse of nursing professionalism appears to have been influenced by ideas and practices from developed countries, but it was unclear from the interviews whether this involved direct contacts or a more general process of knowledge transmission via such communication channels as professional journals and conferences. Few non‐Turkish nationals are employed as nurses in Turkish hospitals, and no nurse in the sample had qualified in a Western country. The one participant who had had a short period of overseas employment (C6) had the opportunity to do so because her surgeon husband took posts in the Netherlands and Belgium to aid his career development. It therefore appears that the professionalising discourse is transmitted mainly through activities and discussions among Turkish nurses themselves, influenced by nursing publications, education and various forms of networking.

### 2.3 Cocooning Nursing Autonomy

With the present medically dominated management structure of Turkish hospitals, changes in practice that might increase nursing autonomy are likely to be limited and dependent on approval from senior doctors. The *professional modernisers* mostly concentrate on bottom‐up initiatives that they hope will change culture through education, team building, nursing guideline development and slowly modifying work culture and patterns of communication.

A participant responsible for nurse education programmes in a MoH hospital sketched out the struggles this involved.

I made a big effort to improve communication and establish a culture of healthy communication. I had a problem finding the money, but more importantly most of the staff didn’t want to join in. They think this is a waste of time. If I make these education sessions compulsory, I need to make staff join in rotation, but then we will have fewer staff in the wards and clinics. It’s a vicious circle. We do everything according to the order that comes from the top. If this kind of education came from top, yes, they would do it. However, this education is mainly on Powerpoint slides, not in practice. Even though it is challenging to organise education, in terms of finding a budget, sponsorship, participants, labour force, I still do it. (…) In Ministry of Health hospitals it is very easy if you want to be transferred to another hospital and city. This is affecting our organisational culture. We educate, train new nurses then they just leave.

(M5‐F‐MoH Hospital)

Several informants mentioned the need to work to change hospital culture and put more emphasis on education and knowledge sharing.

Nowadays most nurses are almost the same age as me, or have a bachelor’s degree at least, especially as the level of education increases. Therefore, our corporate culture is formed to aid information sharing and learning by asking ‘how do you do this? ‘or ‘how did you learn about it?’ We like to share our knowledge and learn new things. Older nurses are the opposite. Our culture begins to turn towards developing a collective movement for more learning. This also increases the quality of communication within the team.

(N19‐F‐MoH Hospital)

Evidence‐based practice was mentioned as an important component of professional development by several participants, and most hospitals have quality units working in this area. However, ensuring that guidelines and protocols that are developed are implemented in routine ward practices can be difficult.

There is a quality unit, which I work in as a nurse, and which is actually responsible for the entire management system of the hospital, that is the nurse's field of practice. For example, a quality standard says what nurses should pay attention to in patient care. (…) Sometimes a very good procedure is prepared, but the nurse doesn't even know that this has been updated, or she doesn't know where to find out about this. She doesn't read the updated information. A deficiency might be found based on an evidence‐based study, for example, changing the assessment of pain. She doesn't know about it, she didn't see it, she didn't learn about it.

(N4‐F‐MoH Hospital)

The interviews gave no indication that Turkish nurses involved in guidance development played the kind of translation role between management, medicine and nursing reported by Spyridonidis and Currie (2016) in the United Kingdom. The process of developing standards and protocols occurred mainly within nursing quality units and nursing teams and did not get nurses a seat in high‐level interdisciplinary management meetings.

When participants reported good nursing teamwork and the ability to make changes to nursing practice, this generally related to work in specialist wards and settings such as intensive care units or emergency departments, where a more self‐contained nursing culture emerges. Although informants reported problems in implementing nursing standards and protocols in general wards, where nursing colleagues might not be fully committed to applying them, this proceeded more smoothly in special settings. Special units typically have a more stable staff group and less intensive oversight by the nursing and medical management hierarchies than do the general wards.

Generally, cooperation, solidarity and information sharing are very high in special units. Frankly, I think that the nurses in the intensive care unit are a little more special and elite, because they have to be. They are engaged in risky work in a high‐risk field. Since care in the unit is high risk, there is a high level of solidarity and knowledge sharing and efficiency.

(C6‐F‐University Hospital)

Interview accounts such as these suggest that the *professional modernisers* had most success in developing nursing autonomy in specialised units that were partially insulated from the wider hospital culture and management structures. In such settings, a well‐motivated staff group aided the operationalisation of the nursing standards the modernisers had devised. These units resemble separate sociocultural worlds, cocoon‐like environments within which teams were subject to less intensive medical direction and are more able to develop their own ideas about effective ways of working and translate these into practice guidelines. They develop a strong sense of in‐group identity, in several cases buttressed by team WhatsApp or email groups and shared social activities.

Organisational memory can be very important for the smooth functioning of specialised units, and nurses rather than doctors tend to be the carriers of such knowledge. A manager with a background in emergency department work explained how this had been important when, some years before, the unit had been required to receive a large number of patients from an aircraft accident.

Corporate memory is very important (…). For example, let me give you a simple example from when there was a plane crash here. (…) We quickly converted an unserviceable clinic into an emergency service and clinic within an hour. In order to take action so quickly [you need experience]. (…) When do you need to do what? Where will you get the material? Where will you get the human resources? How will you create the shift cycle? How will you process these patients? How will you do things? You need an institutional memory and institutional experience for all of these.

(DM6‐F‐University Hospital)

Overall, the positive progress reported by study participants related mainly to small improvements in the settings over which they exercised a measure of control, especially within nursing care teams. Across the hospital as a whole, medical sponsorship in the form of support from a sympathetic medical manager or senior doctor was usually needed, even when suggested innovations involved nursing care procedures.

## 3 Discussion

The study found that nurses in Turkish hospitals are navigating a path that is influenced by aspects of Western discourses on nursing professionalism, but has not, so far, involved significant movement towards interdisciplinary teamwork, hybrid roles or presence in top management. Ideas about the merits of evidence‐based practice, nursing guideline development, teamwork and patient‐centred approaches feature prominently in professional discourses, but concrete examples of change in public hospitals are few, and those that have occurred tend to have low visibility because they are confined within nursing teams rather than wider multidisciplinary teams, and particularly in specialised wards or units. Autonomous action tends to be cocooned within settings where an in‐group nursing culture is strong and medical or line‐management oversight is less intensive.

Most modernisers expressed a preference for a version of nursing leadership involving a flattened nursing hierarchy, continuing professional education, quality improvements in care, evidence‐based practice and care protocols developed with nursing input. However, their professionalising project is more circumscribed and inward‐looking than nursing discourses in countries like the United Kingdom. Turkish hospital nurses recognise the obstacles standing in the way of progress, particularly medical dominance and the civil service culture. They know that their vision of professional development is not well supported by head physicians or indeed most hospital doctors. Moreover, the fact that not all nurses show commitment to this push towards enhanced professionalism means that efforts to introduce nursing innovations often lack ambition and conviction.

Economists studying middle‐ and lower‐income countries have pointed to the importance of the interaction and sequencing of economic policy reforms (Asturias et al. 2016), and the same might be said about the various policy components that make up a wider health reform programme. Health system reform is a complex assemblage of related components, and a successful outcome depends on the coordination and sequencing of change in different system domains. A lesson coming from Türkiye's experience is that the form and feasibility of nursing reform depends on the prior unfolding of reform in related domains. This means that the Turkish nursing profession's ability to traverse a similar path to that taken by nurses in Western countries depends on prior changes in the medical profession and conditions of public‐sector employment, which occurred in the West over an extended time period. Because change in Turkish medicine and the Turkish civil service is constrained by near‐intractable issues of political division, social and gender inequality, traditional hierarchies and patronage, nursing must do its best to chart a way forward in an environment that is largely outside its control. The profession has responded by concentrating on incremental gains in areas where resistance is less strong, while accepting that the more difficult battles must be left for the future (see Inceoz 2024).

The professional discourse conspicuously made no mention of a wish to develop forms of hybrid management that would see nurses assume greater responsibility for audit, performance management or resource allocation. Nor was there any significant incursion of senior nurse managers into territory currently controlled by top hospital managers, who, in Türkiye, are drawn exclusively from the medical profession. The main area of convergence with hybrid professionalism in the West was increased interest in quality improvement and nursing standard development, but this was focused very much on care quality rather than efficiency or economy considerations. Reluctance to become involved with the harder‐edged aspects of general management can be attributed to several factors. NPM reforms have served to further constrain traditional professional work practices and raised tensions several notches higher, especially in areas like PBSPS incentive payments. Moreover, it could be argued that nursing, with a gender balance highly skewed towards a female workforce and the historic association between nursing work and caring domestic roles, finds it even more difficult to adjust to audit and performance management than does medicine (Newman and Lawler 2009). Croft et al. (2015b) highlighted the identity conflict experienced by British nurse leaders when they were called upon to exercise the kind of transactional leadership involved in many hybrid management roles. These senior nurses prefer a version of leadership linked more directly to innovation and quality improvement, an approach closer to the path taken by Turkish nurse modernisers.

Like most qualitative case studies, this one has limitations. The fact that it was carried out during the COVID‐19 pandemic meant, inter alia, that Zoom interviews rather than face‐to‐face interviews were completed, an approach that some critics argue removes the intimacy and rapport of the face‐to‐face interview (O'Connor and Madge 2008). The pandemic also limited the opportunity for methodological triangulation, and we judged that observation or ‘member checking’ (Candela 2019) via a final focus group component would be infeasible. Although the lead author is a native Turkish speaker, the study threw up difficult translation issues in conveying the tone and nuances of nurses' accounts, because different language idioms mean that simple word‐to‐word translation is not possible. The original plan for the study tentatively explored the possibility of recruiting doctors as a comparison group, but although this would have undoubtedly resulted in a more comprehensive study, access and logistical problems rendered the idea infeasible. Finally, we must acknowledge that this is a case study of nurses in a limited number of Istanbul public‐sector hospitals, meaning that the generalisability of the findings cannot be assumed.

The Turkish experience shows that different approaches to the professional development of nursing exist in different countries so that no single global trajectory of change has emerged. Türkiye's ‘in‐between’ status as a rising upper‐middle‐income country with close economic ties to the Western countries of the European Union makes it an interesting case study because it has charted a development path that several middle‐ and lower‐income countries may follow. Nurses in many such countries with healthcare systems subject to a substantial degree of medical control may also develop nursing practice in ways that deliver at least some gains while not directly challenging existing organisational structures and distributions of power. Very little research currently exists on the extent to which the rise of ideas such as interdisciplinary working and distributed leadership in Western countries has changed practice in countries with very different cultures and healthcare systems. This paper makes a start in filling this knowledge gap, based on what we believe to be the only major qualitative study of Turkish hospital nurses carried out in recent years and at a juncture when the modernisation of nurse management structures is a pressing policy issue for the Ministry of Health and the Turkish Nursing Association.

## Author Contributions

**Zuleyha Inceoz:** conceptualization, data curation, formal analysis, writing – original draft, methodology, investigation, writing – review and editing. **David Hughes:** conceptualization, formal analysis, methodology, supervision, writing – original draft, writing – review and editing.

## Data Availability

Research data are not shared.

## Endnotes

^1^
Interview extracts are labelled using the following notation. The first component, Mx, Sx, DMx, Cx and Nx, refers to nurse grade and identifying number. The categories used are senior nurse managers (M), supervisor nurses (S), deputy manager nurses (DM), charge nurses (C) and staff nurses (N). The number that follows (‘x’) identifies the participant. M or F refers to male or female gender. Lastly, MoH, university or city hospital identifies hospital type.
